# Invasive Pulmonary Aspergillosis in an (Apparently) Immunocompetent Patient

**DOI:** 10.7759/cureus.10238

**Published:** 2020-09-04

**Authors:** Francisco Teixeira da Silva, Miguel Romano, Alexandra Esteves, José Carvalho, Manuel Ferreira

**Affiliations:** 1 Internal Medicine, Unidade Local de Saúde do Alto Minho, Viana do Castelo, PRT

**Keywords:** invasive aspergillosis, copd, immune response

## Abstract

Invasive pulmonary aspergillosis (IPA) is an opportunistic infection that usually threatens immunocompromised patients. However, there are some reports of IPA in immunocompetent patients without the obvious classic risk factors. We present the case of an 82-year-old woman with a prior medical history of chronic obstructive pulmonary disease (COPD) and a recent short-term corticosteroid regimen for an acute exacerbation. She was admitted with dyspnoea, cough, and pleuritic pain and was diagnosed with pneumonia. Clinical deterioration occurred, and a diagnosis of IPA was made. She received treatment with voriconazole but died 14 days after admission. This case highlights the importance of considering IPA among the possible causes of infection in this population. Prompt institution of appropriate antifungal therapy is paramount for the management of this condition.

## Introduction

Aspergillus spp. are ubiquitous fungi found in the soil, rubble, and indoor environment. They may cause infectious or allergic diseases depending on the host’s immune situation and pulmonary structure [1]. Invasive pulmonary aspergillosis (IPA) occurs most frequently in patients receiving long-term corticosteroids and in transplant and oncologic patients, predominantly those with haematological disease. Aspergillosis may, in rare situations, occur in immunocompetent individuals [2].

## Case presentation

We present the case of an 82-year-old female nursing-home resident with a previous medical history of valvular heart failure and chronic obstructive pulmonary disease (COPD). One month before her admission, she was diagnosed with infected bronchiectasis. She received in-hospital treatment with levofloxacin and systemic corticosteroids (methylprednisolone) during 14 days and recovered completely. She presented to our emergency department with a two-day history of worsening dyspnoea, productive cough, and pleuritic pain. On clinical examination, she was tachypneic and hypotensive, with decreased breathing sounds and bibasal crackles on pulmonary auscultation. Laboratory workup showed leucocytosis with neutrophilia, hypoxia (pO_2_ 51 mmHg - fiO_2_ 0.21), and elevation of C-reactive protein (CRP) and brain natriuretic peptide (BNP) (Table 1).

**Table 1:** Laboratory workup Htc, haematocrit; CRP, C-reactive protein; BNP, brain natriuretic peptide; HBV, hepatitis B virus; HCV, hepatitis C virus

Tests	Reference values	Results
Haemoglobin (g/dL)	11.8-15.8	12
Htc (%)	36-46	35.7
Leuocytes (µL)	4.0-10.0	21.670
Neutrophils	1,800–7,700	19.280 (89%)
Lymphocytes	800-4,000	910 (4.2%)
Platelets (10^9^/µL)	150-400	220
Glucose (mg/dL)	70-110	175
Urea (mg/dL)	17-43	90
Creatinine (mg/dL)	0.6-1.0	0.81
Sodium (mmol/L)	136-145	131
Potassium (mmol/L)	3.5-5.1	5.4
CRP (mg/dL)	0.01-0.82	3.34
BNP (pg/mL)	<100	334.5
HBV, HCV, HIV1-2 serologies		Negative

**Figure 1:**
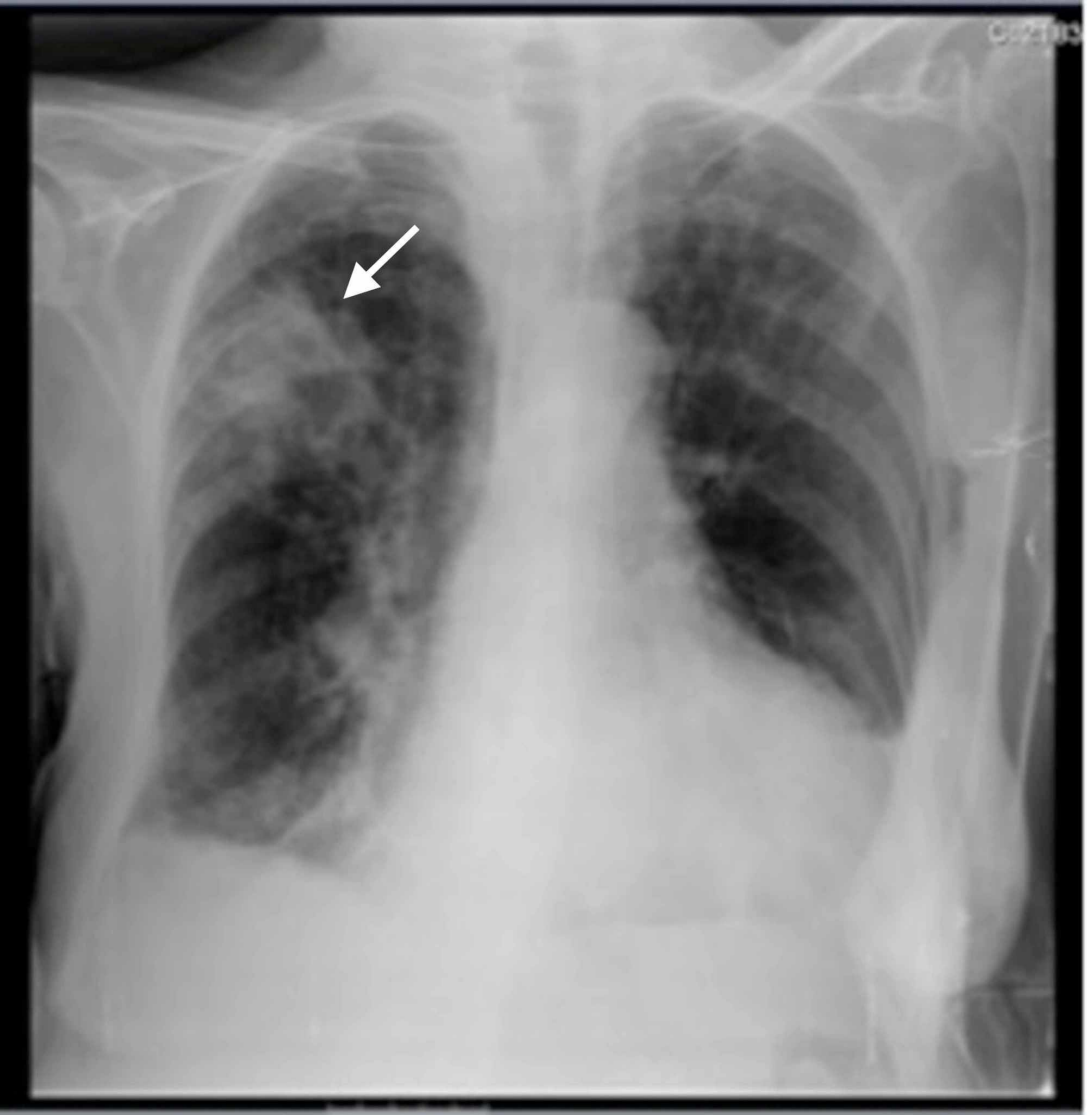
Chest X-ray Condensation image in the right upper lobe (arrow).

A chest X-ray revealed an image of condensation in the right upper lobe (Figure 1). A diagnosis of nosocomial pneumonia was made and she started empirical antibacterial therapy with piperacillin-tazobactam. She continued with persistent fever, shortness of breath, and hypoxaemia and did not respond to initial therapy with inhaled bronchodilators plus systemic corticosteroids. 

**Figure 2:**
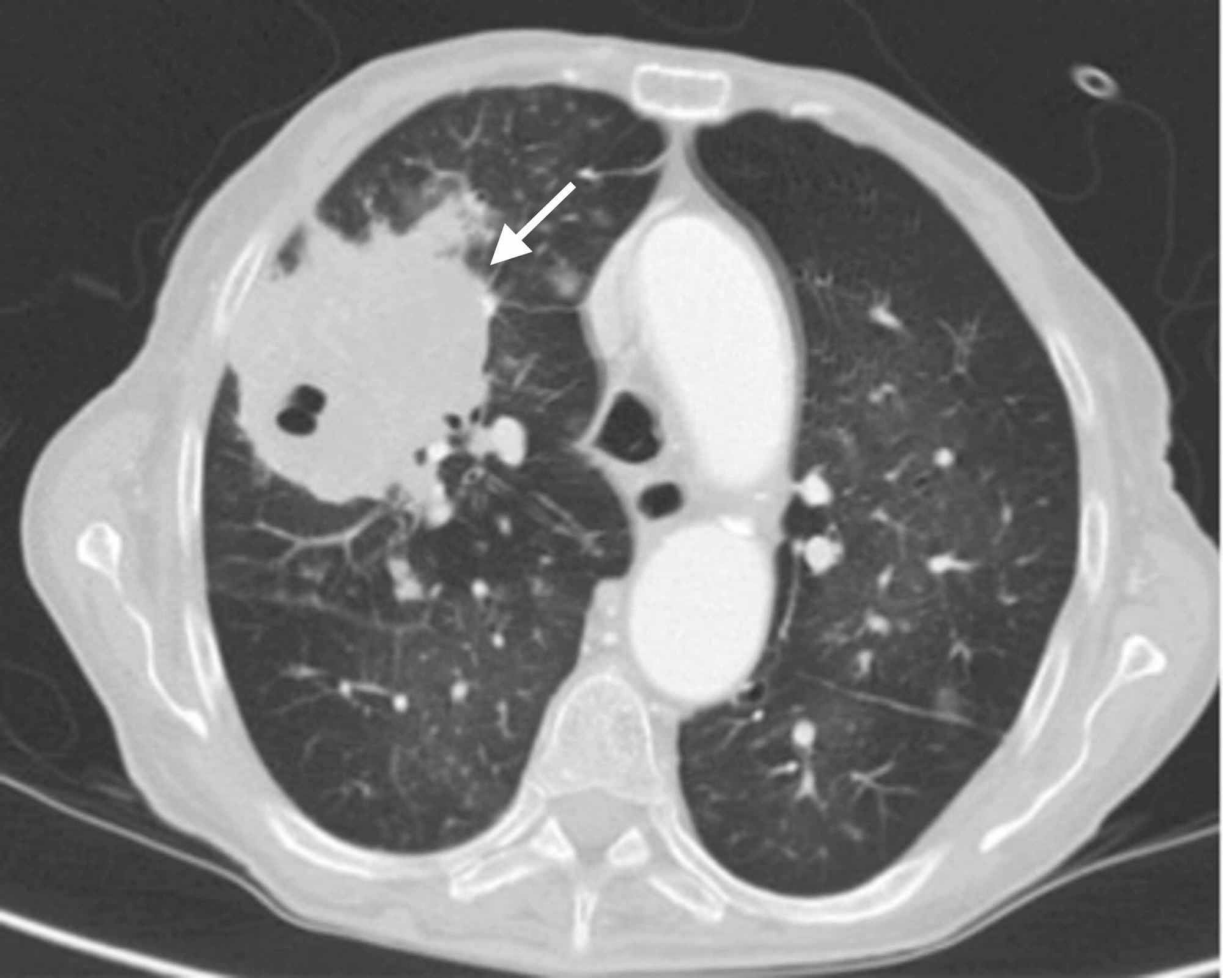
CT scan Areas of consolidation in the right upper lobe with gas-fluid levels inside, suggesting necrotizing pneumonia (arrow).

A chest CT scan revealed areas of consolidation in the right upper lobe with gas-fluid levels inside, indicating necrotizing pneumonia and several scattered micronodules suggesting endobronchial dissemination of an acute infective aetiology (Figure 2). Sputum samples for acid-fast bacilli (AFB) were negative, and the patient was evaluated for eosinophilia but peripheral blood smear did not show any relevant abnormalities. Antibiotics were escalated and the patient was started on imipenem and clindamycin. A bronchoscopy was performed and the bronchoalveolar lavage (BAL) fluid was negative for AFB and bacterial pathogens. BAL cytology showed hyphae, and numerous colonies of Aspergillus fumigatus were found on culture examination. The patient was managed with voriconazole but developed multiorgan failure and died on hospital day 14. 

## Discussion

In most cases, infectious Aspergillus spores are introduced to the lower respiratory tract by inhalation. They can germinate into hyphae and can then invade the mucosa leading to IPA [3]. The diagnosis remains difficult; symptoms, which include fever unresponsive to antibiotics, productive cough, and dyspnoea, are non-specific and usually mimic pneumonia [4].

Tissue biopsy with histopathologic demonstration of tissue invasion by fungal hyphae is the ‘gold standard’ but is not always possible. Regarding imaging exams, a chest CT scan is recommended for the evaluation of lung parenchyma. Pulmonary nodules are the most commonly identified abnormalities, and the historic ‘halo sign’ is fairly specific for pulmonary aspergillosis. Laboratory tests to identify antigens in body fluids (e.g. serum or BAL) are also helpful. Galactomannan (GM) testing or Aspergillus polymerase chain reaction (PCR) assay in BAL has decent specificity and sensitivity for IPA [4].

The revised EORTC/MSG criteria define invasive fungal disease and were designed to advance clinical and epidemiological research as well as to help and guide clinicians in daily practice [5]. The isolation of Aspergillus spp. from a sterile BAL fluid from immunosuppressed patients is highly indicative of invasive aspergillosis.

Classic risk factors for API include prolonged neutropenia, transplantation (the highest risk with lung transplant and haematopoietic stem-cell transplantation), chemotherapy, immunosuppression, and neoplasia (namely haematological). Several recent studies reinforce that the host's immune status and response play a fundamental role in the onset and evolution of the disease. Critical and COPD patients should therefore also be considered at risk [1,2].

It is not completely established why COPD patients may develop IPA. Potential risk factors predisposing COPD patients include corticosteroid therapy, the severity of underlying lung disease and COPD staging, critical illness, previous colonization of airways by Aspergillus spp., impaired mucociliary clearance, frequent use of broad-spectrum antibiotics, and possibly genetic factors (such as surfactant proteins or mannose-binding protein defects) [6].

Despite the introduction of several new antifungal agents, the treatment of IPA remains difficult and mortality rates are still high. Voriconazole is the first-line treatment of IPA, and alternative agents include caspofungin, itraconazole, and amphotericin B. Treatment duration should be individualized to clinical and radiological response. The treatment is often lengthy, lasting from several months to up to one year. Surgical resection has a very limited role in the management of such patients but should be considered in cases with extrapulmonary involvement, such as bone invasion, burn victims, epidural abscesses, and ocular disease [1,2].

## Conclusions

This case illustrates that COPD, short-term corticosteroid, and older age should be considered as risk factors for IPA. The diagnosis is often delayed due to lack of suspicion. When presented with worsening or refractory symptoms and increasing pulmonary abnormalities on imaging studies, investigation to rapidly confirm the diagnosis is warranted.
